# Supplementary material for: Patterns of mammalian jaw ecomorphological disparity during the Mesozoic/Cenozoic transition
Source: Proc Biol Sci. 2019 May 1;286(1902):20190347. doi: 10.1098/rspb.2019.0347 (PMC6532522; doi:10.1098/rspb.2019.0347)
Supplement: Benevento_etal_functional_traits_methods_results_ESM [file rspb20190347supp1.pdf]

Measurements Collected
The following ten continuous character measurements were collected and used to produce six ratios that serve as the mechanical traits used in this study.

CM1: Jaw length
Maximum length of the jaw, from the posterior most point of the jaw to the tip of the tooth row. The most posterior point on the jaw may consist of the angular, articulation, or coronoid. The total size of the jaw directly relates to the maximum size of food items that can be consumed.

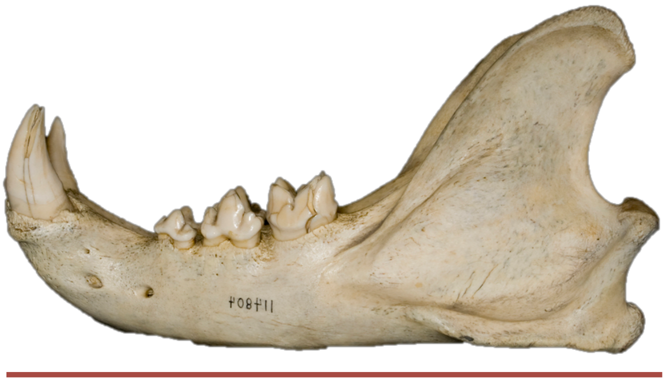

Figure CM1: ***Panthera leo*** **jaw, with red line indicating jaw length measure**

CM2: Jaw depth
Depth of the jaw immediately posterior to the last (most posterior) tooth, usually the last molar.

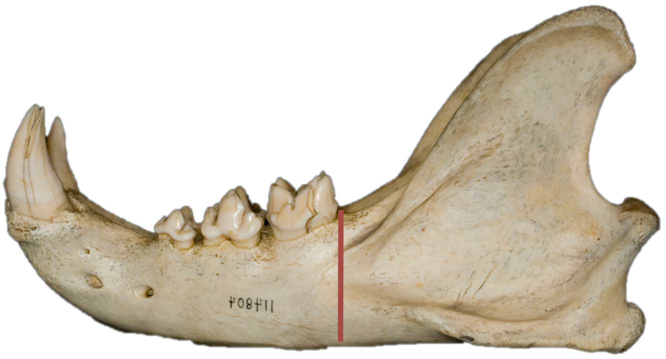

Figure CM2: ***Panthera leo*** **jaw, with red line indicating jaw width measure**

CM3: Dental row length

Total length of the tooth row, from the back of the last tooth, often the ultimate molar, to the anterior margin of the most anterior tooth, often the first incisor. The length of the tooth row is the total length of the area where food can be processed.

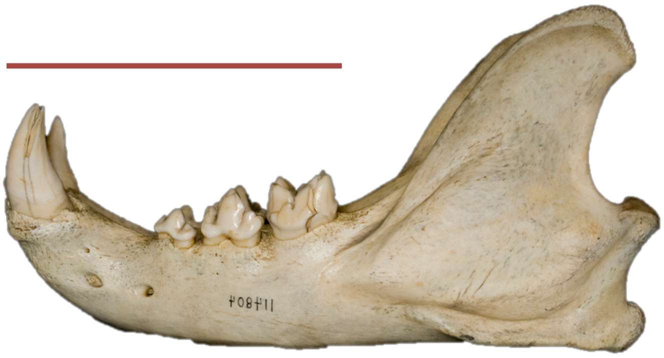

Figure CM3: ***Panthera leo*** **jaw, with red line indicating dental row length measure**

CM4: Molar row length

Total length of the molar row, from the posterior margin of the last molar to the anterior margin of the first premolar.

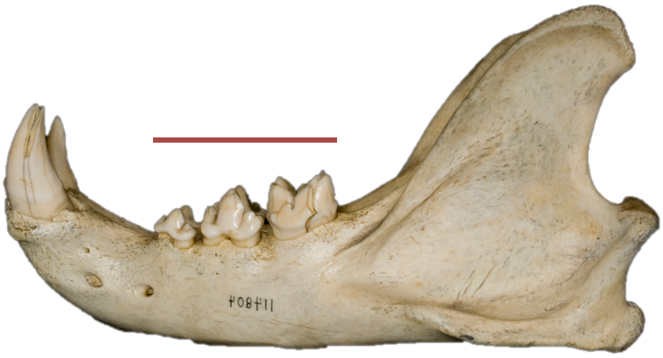

Figure CM4: ***Panthera leo*** **jaw, with red line indicating molar row length measure**

CM5: Diastema length
Total length of the diastema.

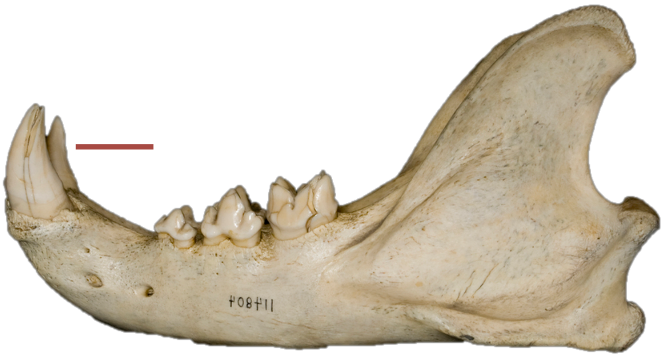

Figure CM5: *Panthera leo* jaw, with red line indicating diastema length measure

CM6: Distance from condyle to coronoid
Total distance from the most posterior surface of the condyle, to the tallest point on the margin of the coronoid.

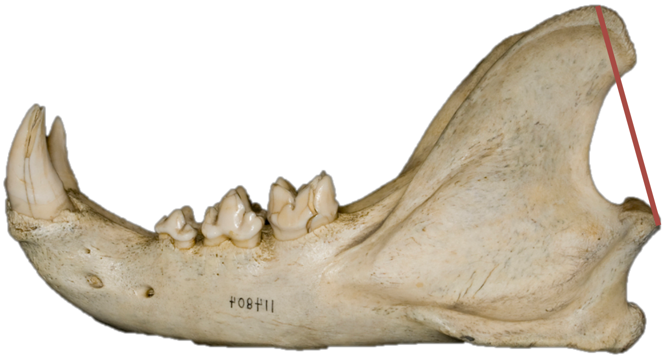

Figure CM6: *Panthera leo* jaw, with red line indicating condyle to coronoid measure

CM7: Distance from condyle to middle cusp of last premolar
Total distance from the most posterior margin of the condyle to the middle cusp of the last premolar.

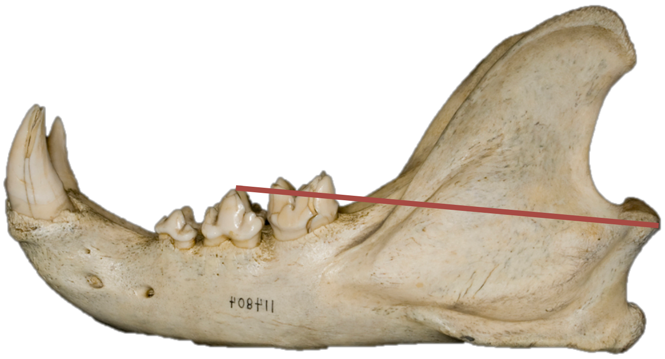

Figure CM7: *Panthera leo* jaw, with red line indicating condyle to premolar cusp measure

CM8: Coronoid length

Total length of the coronoid, from the base of the coronoid where the trough positioned anteriorly to the condyle curves upward, to the highest point on the dorsal margin of the coronoid.

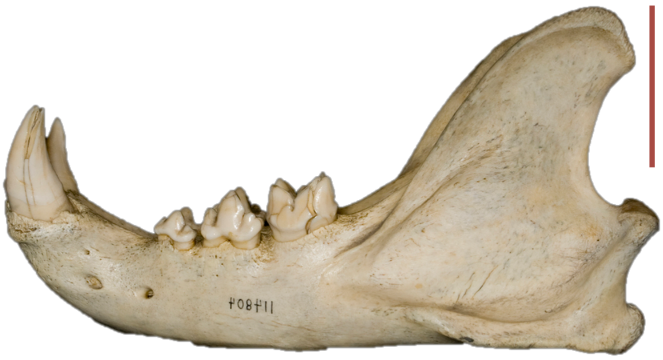

Figure CM8: ***Panthera leo*** **jaw, with red line indicating coronoid length measure**

CM9: Coronoid width

Total width of the coronoid from the posterior margin to the anterior margin, in line with the point where the trough positioned anteriorly to the condyle curves upward.

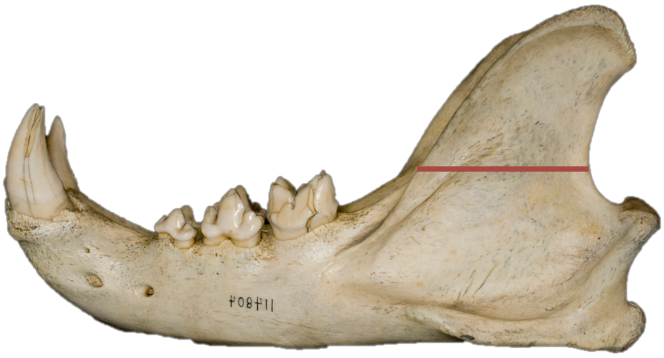

Figure CM9: ***Panthera leo*** **jaw, with red line indicating coronoid width measure**

CM10: Distance from articulation to tooth row

The articular offset of the jaw is measured in a vertical line from the highest dorsal point of the articulation, the condyle, to a line drawn along the top surface of the tooth row. This line is drawn from the dorsal surface of the most proximal tooth, usually the first incisor, to the dorsal surface of the most distal tooth, usually the last molar, and extended backwards along the length of the jaw (see grey line). The cusps of teeth between the most proximal and most distal teeth may fall above or below this line. The canine often extends above this line, however the line best captures the occlusional surface of the teeth. The vertical line measured may extend above or below the grey tooth row line (see figures 15a and 15b). In mammals, an articulation positioned above the tooth row is more frequently observed.

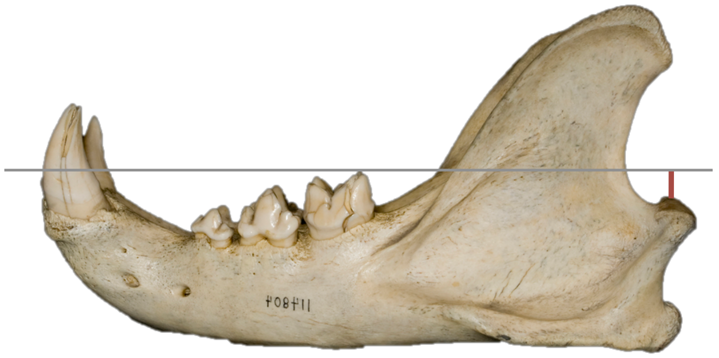

Figure CM10a: ***Panthera leo*** **jaw, with grey line indicating the occlusional surface of the tooth row and red line indicating articulation offset measure**

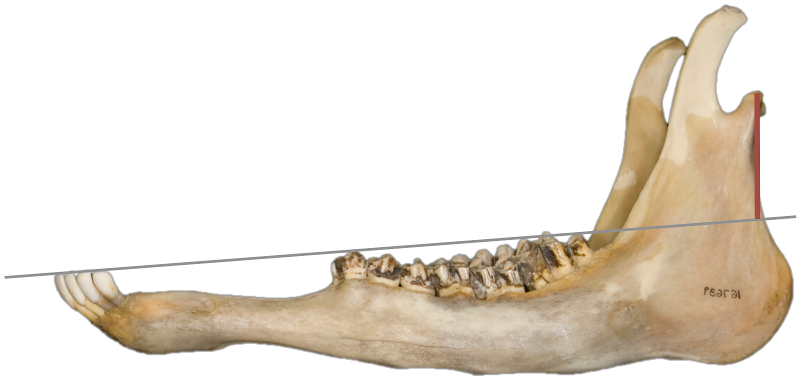

Figure CM10b: ***Boselaphus tragocamelus*** **jaw, with grey line indicating the occlusional surface of the tooth row and red line indicating articulation offset measure**

Traits used

The following ratios are used to capture key functional traits across mammalian jaws, and to reflect their biomechanical properties. An organism's biomechanics defines and confines it's functional abilities, and therefore can help to discriminate between different ecomorphologies.

Where appropriate (indicated in following text), proportional ratios have been arc sine transformed. This spreads out the tails of the distribution for proportions.

All data has then been z transformed. This scales all traits so that their mean is equal to 0, and allows each trait to carry equal weight in the PCA analysis.

T1: Diastema length ratio

The diastema, the space between teeth near the front of the tooth row, has clear functional relevance and appears to be a good indicator of ecology. The diastema can be between the canine and first premolar, between the first and second premolars, or between the incisor and first premolar (where a canine is not present). A diastema positioned behind the canine is more usual in mammals. Herbivorous mammal species, including species within clades like Artiodactyla, have a large diastema. This diastema serves as a holding space for food that has been picked/grazed before it is passed backwards so that mastication can commence on the premolar/molar teeth. Hypercarnivores also have a relatively large diastema, however this space is likely not used as a holding area for torn off flesh. The diastema in hypercarnivores allows space for the upper canine to come into line with the lower canine, and allows the canines to pierce further into carrion. Diastema length is divided by tooth row length to capture how large the diastema is relative to the overall size of the tooth row.

The diastema length ratio acts as a proportion and is therefore arc sine transformed.

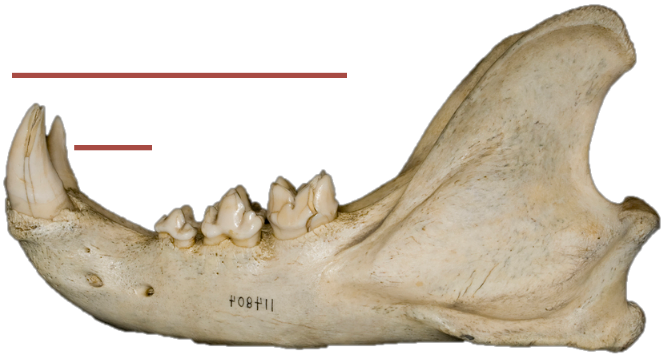

Figure T1: ***Panthera leo*** **jaw, with red lines indicating diastema length ratio (diastema length/dental row length)**

T2: Molar row length ratio

The molar row length ratio describes the amount of the tooth row that has been specialised for mastication. Browsers and grazers should have a larger proportion of their tooth row dedicated to this function. The molar row length ratio is calculated by dividing molar row length by dental row length.

The molar row length ratio acts as a proportion and is therefore arc sine transformed.

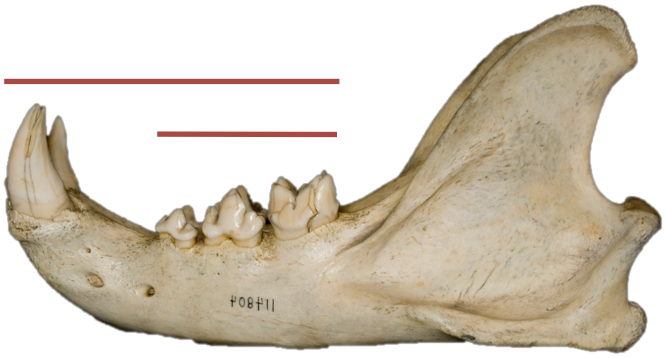

Figure T2: *Panthera leo* jaw, with red lines indicating molar row length ratio (molar row length/dental row length)

T3: Jaw closing mechanical advantage

The mammalian jaw can be simplified as a 2D third-order leaver. The in-leaver and out-leaver both originate from the condyle, which acts as the fulcrum (the point of rotation). The in-leaver is measured from the posterior margin of the condyle to the highest marginal point of the coronoid. The out-leaver for average jaw closing mechanical advantage is measured from the posterior margin of the condyle to the middle cusp of the last premolar. Force, provided by the m. temporalis muscle complex, is applied to the in-leaver in order to lift the out-leaver

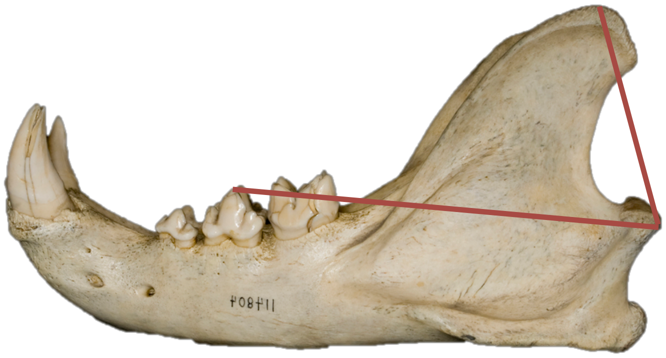

Figure T3: *Panthera leo* jaw, with red lines indicating average jaw closing mechanical advantage measure (condyle to coronoid/condyle to premolar)

T4: Jaw slenderness ratio

This describes the stiffness of the jaw. The jaw depth measure can serve as a proxy for flexural stiffness under dorso-ventral loads [1].
The jaw slenderness ratio is calculated by dividing jaw depth by jaw length *100.

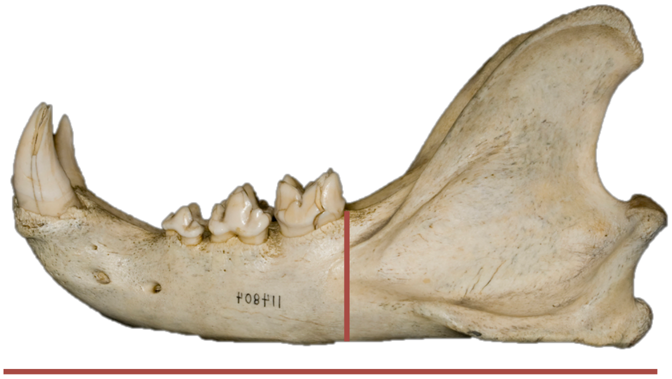

Figure T4: ***Panthera leo*** **jaw, with red lines indicating jaw slenderness ratio (jaw depth/jaw length * 100)**

T5: Coronoid slenderness ratio

The temporalis muscle attaches to the coronoid in two places. The m. temporalis pars superficialis extends from the temporal fossa on the skull to the lateral surface of the coronoid process. The m. temporalis pars profunda extends from the anterior portion/margin of the temporal fossa on the skull to the medial surface of the coronoid process on the jaw. Another muscle, the m. masseter pars profunda attaches from the medial surface of the zygomatic arch to the lower jaw along the lower margin of, or immediately below, the coronoid process (but see wombat musculature [2]). These muscles play a role in jaw opening and closing mechanics and bite force. Differences in the size and shape of the condyle, and therefore the surface area of the muscle attachment sites, are indicative of the strength of the organisms bite. Further, the more anterioposteriorally wide the coronoid process, the more resistant it is to forces, whereas more anterioposteriorally narrow coronoid processes will withstand less force.
Coronoid length is divided by coronoid width and then times by 100 to describe the slenderness of the coronoid process, therefore capturing the robustness of the coronoid and therefore a relative measure of how much tension it can take.

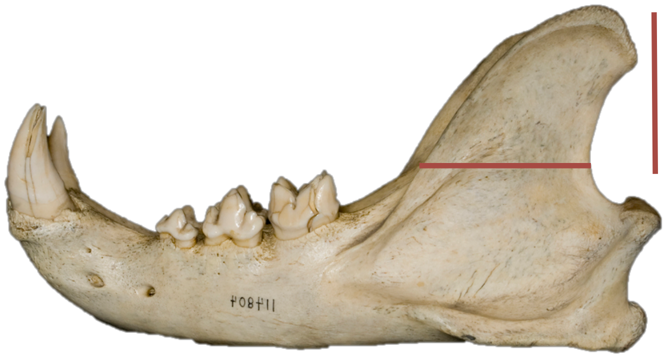

Figure T5: ***Panthera leo*** **jaw, with red lines indicating coronoid slenderness ratio ((coronoid length/coronoid width)*100)**

T6: Articulation offset ratio.

The position of the articulation relative to the tooth row affects the way in which the upper and lower teeth occlude. When the articulation is in line with the tooth row (i.e. a measurement of 0), the jaw closes in a scissor-like movement. As the articulation moves further away from the tooth row line occlusion occurs in a flatter, up and down motion, allowing all teeth to occlude at once. Broadly, carnivores have a more scissor-like occlusion to allow for a slicing action, and herbivores occlude their teeth all at once to allow for grinding of plant material.

Articulation offset ratio is calculated by dividing the distance between the jaw joint and the tooth row by jaw length.

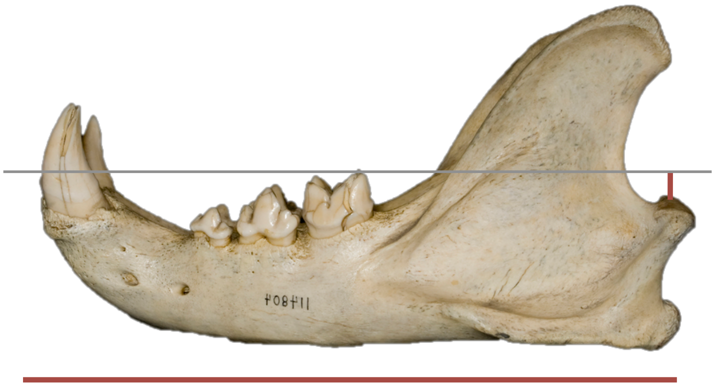

Figure T6a: *Panthera leo* jaw, with grey line indicating the occlusional surface of the tooth row, vertical red line indicating articulation offset measure, and horizontal red line indicating jaw length. Articulation offset ratio (distance between the jaw joint and the tooth row/jaw length)

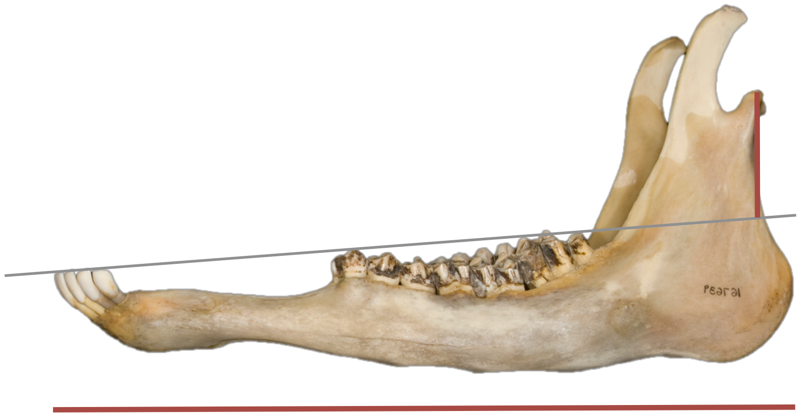

Figure T6b: *Boselaphus tragocamelus* jaw, with grey line indicating the occlusional surface of the tooth row, vertical red line indicating articulation offset measure, and horizontal red line indicating jaw length. Articulation offset ratio (distance between the jaw joint and the tooth row/jaw length)

1. Anderson PSL, Friedman M, Ruta M. 2013 Late to the Table: Diversification of Tetrapod Mandibular Biomechanics Lagged Behind the Evolution of Terrestriality. *Integr. Comp. Biol.* **53**, 197–208. (doi:10.1093/icb/ict006)

2. Sharp AC, Trusler PW. 2015 Morphology of the Jaw-Closing Musculature in the Common Wombat (*Vombatus ursinus*) Using Digital Dissection and Magnetic Resonance Imaging. *PLOS One* **10**, e0117730. (doi:10.1371/journal.pone.0117730)

Supplementary Methods

Trait Collection (extended)

The 10 measurements outlined above were collected from fossils mammal mandibles from the Late Triassic-Eocene. These measurements were taken from mandible images in published literature, and first hand from museum and university collections. GLB visited the following 8 collections between May 2015 and April 2017 to collect mandible measurement data; OUMNH, Naturmuseum Senckenburg, Hessisches Landesmuseum Darmstadt, AMNH, Field Museum, Carnegie Museum, Smithsonian Institution NMNH, and Johns Hopkins University (Collections curated and collected by Ken Rose).

Only terrestrial mammals were included in this study, and mammals were included at species level where possible. Where specimens identified to species level were not available, one example of the genus is included instead.

Measurements were taken from complete jaws and reasonable partial jaws. Where, for example, the anterior tip of the tooth row, or the tip of the coronoid process are missing, measurements are estimated by GLB based on knowledge of the anatomy of closely related species.

Taxonomic Affiliations

Many of the older mammal clades included in this study have no living descendants or are basally branching members of living clades. Therefore phylogenetic placement and taxonomic affiliation can be challenging and sometimes multiple hypotheses for one clade or genus can exist in the literature. Where this is the case, the affiliation/placement preferred by the authors or the more commonly accepted affiliation/placement are used, and we outline these decisions below.

Of particular note is the placement of the clade Haramiyida. Two competing hypotheses exist. Haramiyids have been recovered as non-mammalian mammaliaforms [e.g 1, 2], and alternatively within Allotheria, as a sister clade to multituberculates [3] or as a paraphyletic group with the monophyletic multituberculates deriving from 'haramiyidans' [e.g. 4-7]. For the purpose of this study, we follow the phylogenetic placement of Luo et al [1] and assign haramiyids and euharamiyids to non-mammalian mammaliaforms. We use this placement mainly because the research efforts of Luo et al [1] were aimed primarily at addressing the discrepancies in haramiyid placement in the mammalian tree, and concluded that the clade fell within non-mammalian mammaliaforms based on detailed anatomical study. It should be noted however that truly conclusive evidence for the placement of this clade is lacking, and both hypotheses are likely to remain valid until more conclusive anatomical evidence can be found in the fossil record. Despite this, the assignment of haramiyids to non-mammalian mammaliaforms in this study does not affect our overall conclusions regarding the timings and magnitude of the ecomorphological radiation of mammals.

A consensus on the placement of the species *Eomaia scansoria* and *Juramaia sinensis* is also lacking. Across published phylogenies, these two taxa jump between positions among the most basally branching eutherian mammals [e.g. 8-10] and positions within stem Theria [e.g. 11]. Here, we follow a eutherian

placement of these taxa [8-10]. Including these contested species within Eutheria does not affect the disparity curves produced for therian mammals, as both *E. scansoria* and *J. sinensis* fall within time bins that pre-date bins that include sufficient numbers of therian mammals to allow for disparity to be calculated.

Finally, *Pseudotribos robustus* is here included as an Australosphenida, and is affiliated with the monotreme stem [e.g. 12, 13] (labelled Australosphenida-Prototheria-Monotremata in Figs. 1, S1-S5). This is in contrast to hypotheses placing either all members of Australosphenida or a subset of species which have otherwise been affiliated with Australosphenida closer to eutherians than to monotremes [14-16]. Once again, the placement of *Pseudotribos robustus* within Australosphenida does not affect our overall conclusions regarding mammal jaw disparity through time.

Placement of Taxa into Time Bins

Species range dates were collected for each taxa, and specimens were placed in all bins that the species is known from. For some taxa, precise dates for the formations that they have been found in are unknown. In these instances, an age range may be given, representing the window of time during which the species may have lived. For such taxa, rather than placing the species into all bins within their age range, we randomize the bin with every bootstrap sample.

When plotting function space through time, these taxa are represented as full-size symbols in the most likely bin (assigned by GLB based on additional data on probable ages and/or the amount of overlap with any given bin), and at smaller sizes in other possible—but less likely—intervals.

References

1. Luo Z-X, Gatesy SM, Jenkins Jr FA, Amaral WW, Shubin NH. 2015 Mandibular and dental characteristics of Late Triassic mammaliaform Haramiyavia and their ramifications for basal mammal evolution. *PNAS* **112**, E7101-E7109. (doi:10.1073/pnas.1519387112)

2. Huttenlocker AK, Grossnickle DM, Kirkland JI, Schultz JA, Luo Z-X. 2018 Late-surviving stem mammal links the lowermost Cretaceous of North America and Gondwana. *Nature* **558**, 108-112. (doi:10.1038/s41586-018-0126-y)

3. Kielan-Jaworowska Z, Cifelli RL, Luo Z-X. 2004 *Mammals from the Age of Dinosaurs: Origins, Evolution, and Structure.* New York: Columbia Univ. Press.

4. Butler PM. 2000 Review of the early allotherian mammals. *Acta. Palaeontol. Pol.* **45**, 317-342.

5. Hahn G, Hahn R. 2006 Evolutionary tendencies and systematic arrangement in the Haramiyida (Mammalia). *Geol. Palaeontol.* **40**, 173-193.

6. Averianov AO, Lopatin AV. 2011 Phylogeny of triconodonts and symmetrodonts and the origin of extant mammals. *Dokl. Biol. Sci.* **436**, 32-35. (doi:10.1134/S0012496611010042)

7. Meng J, Bi S, Zheng X, Wang X. 2018 Ear ossicle morphology of the Jurassic euharamiyidan *Arboroharamiya* and evolution of mammalian middle ear. *Journal of Morphology* **279**, 441-45. (doi:10.1002/jmor.20565)

8. Ji Q, Luo Z-X, Yuan C-X, Wible JR, Zhang J-P, Georgi JA. 2002 The earliest known eutherian mammal. *Nature* **416**, 816–822. (doi:10.1038/416816a)

9. Wible JR, Rougier GW, Novacek MJ, Asher RJ. 2009 The eutherian mammal *Maelestes gobiensis* from the Late Cretaceous of Mongolia and the phylogeny of Cretaceous Eutheria. *Bull. Am. Mus. Nat. Hist.* **327**.

10. Luo Z-X, Yuan C-X, Meng Q-J, Ji Q. 2011 A Jurassic eutherian mammal and divergence of marsupials and placentals. *Nature* **476**, 442–445. (doi:10.1038/nature10291)

11. O'leary MA et al. 2013 The Placental Mammal Ancestor and the Post–K-Pg Radiation of Placentals. *Science* **339**, 662-667. (doi:10.1126/science.1229237)

12. Rauhut OWM, Martin T, Ortiz-Jaureguizar E, Puerta P. 2002 A Jurassic mammal from South America. *Nature* **416**, 165–168. (doi:10.1038/416165a)

13. Luo Z-X, Ji Q, Yuan C-X. 2007 Convergent dental adaptations in pseudo-tribosphenic and tribosphenic mammals. *Nature* **450**, 93–97. (doi:10.1038/nature06221)

14. Rich TH, Flannery TF, Trusler P, Kool L, Van Klaveren, Patricia Vickers-Rich P. 2002 Evidence that monotremes and ausktribosphenids are not sistergroups. *J. Vertebr. Paleontol.* **22**, 466-469. (doi:10.1671/0272-4634(2002)022[0466:ETMAAA]2.0.CO;2)

15. Woodburne MO, Rich TH, Springer M. 2003 The evolution of tribospheny in Mesozoic mammals. *Mol. Phylogenet. Evol.* **28**, 360–385.

16. Rich TH, Vickers-Rich P. 2013 Palaeobiogeography of Mesozoic Mammals – Revisited. *Earth and Life: Global Biodiversity, Extinction Intervals and Biogeographic Perturbations through Time*, ed Talent JA. New York: Springer. pp 913-934.

Principal Components

PC1 describes 32.78% of overall variance, and correlates with (in order of significance) the length of the diastema in comparison to the length of the tooth row, the length of the molar row in comparison to the length of the tooth row, the slenderness of the jaw, and to a lesser extent the articulation offset in comparison to the length of the jaws. Mammals plotting against more positive PC1 scores have particularly slender, shallow jaws, little or no diastema and a long relative molar row, with a low articulation offset ratio. Mammals that exhibit more negative PC1 scores have an opposite suite of traits.

PC2 describes 25.34% of overall variance, and correlates with the following traits, in order of significance (SI Table 1); the articulation offset in comparison to the length of the jaw, jaw closing mechanical advantage, coronoid slenderness, and to a lesser extent the length of the molar row in comparison to the length of the tooth row. Mammals that plot more positively on PC2 have lower scores for jaw closing mechanical advantage, more anteroposteriorly slender coronoids, a larger relative articulation offset, and a longer relative molar row length.

PC3 describes 17.6% of overall variance. In order of significance, PC3 correlates well with jaw slenderness and jaw closing mechanical advantage, and to a lesser extent the length of the molar row in comparison to the length of the tooth row. Mammals plotting more positively on PC3 have more slender, shallower jaws, a smaller mechanical advantage, and a larger molar row in comparison to the tooth row.

PC4 describes 11.72% of the variation, and correlates primarily with coronoid slenderness ratio. Mammals that plot in the most positive positions on PC4 have coronoid processes which are more slender anterioposteriorally and more elongate dorsoventrally.

PC5 describes 6.91% of the variance. PC5 does not correlate strongly with any trait in particular, but correlates with the articulation offset ratio, jaw slenderness, and jaw closing mechanical advantage the most. Generally speaking, mammal species which plot more positively on PC5 have a larger relative articulation offset, a more slender jaw, and a larger jaw closing mechanical advantage.

PC6 describes 5.61% of the overall variance, but again does not correlate well with any individual trait. In order of significance, PC6 correlates best with diastema length, and relative molar length. Mammals plotting more positively on PC6 have, on average, larger relative diastemas, and longer relative molar row.

PC axis	Trait	Correlation	p-value
PC1	diastema.logit	0.787106304	3.18E-55
PC1	jaw.slenderness	0.655388	8.35E-33
PC1	articulation.offset.ratio	0.588886247	2.74E-25
PC1	molar.logit	-0.5804621	1.86E-24
PC1	coronoid.slenderness	0.459780565	8.54E-15
PC1	closing.ma.premolar	0.151992837	0.014927096
PC2	closing.ma.premolar	0.661647429	1.30E-33
PC2	articulation.offset.ratio	-0.619182188	1.72E-28
PC2	molar.logit	-0.55719545	2.79E-22
PC2	coronoid.slenderness	-0.547330781	2.08E-21
PC2	diastema.logit	0.293272616	1.80E-06
PC2	jaw.slenderness	-0.058825161	0.348548453
PC3	jaw.slenderness	0.628173055	1.65E-29
PC3	closing.ma.premolar	0.566865229	3.65E-23
PC3	molar.logit	0.449036217	4.17E-14
PC3	diastema.logit	-0.309899428	4.21E-07
PC3	coronoid.slenderness	-0.157046349	0.011866934
PC3	articulation.offset.ratio	0.134020148	0.03207236
PC4	coronoid.slenderness	0.6724674	4.71E-35
PC4	closing.ma.premolar	0.375429307	5.44E-10
PC4	articulation.offset.ratio	-0.232731519	0.000171824
PC4	jaw.slenderness	-0.183756987	0.003168577
PC4	diastema.logit	-0.149590628	0.01660996
PC4	molar.logit	-0.015467393	0.805464013
PC5	articulation.offset.ratio	0.441749232	1.18E-13
PC5	jaw.slenderness	-0.309758555	4.26E-07
PC5	closing.ma.premolar	0.23994336	0.000105711
PC5	diastema.logit	-0.172695778	0.005597786
PC5	molar.logit	-0.158296512	0.011201112
PC5	coronoid.slenderness	-0.107775043	0.085254827
PC6	diastema.logit	-0.382382002	2.45E-10
PC6	molar.logit	-0.354494612	5.39E-09
PC6	jaw.slenderness	0.206610238	0.000882474
PC6	closing.ma.premolar	-0.138797295	0.026374812
PC6	articulation.offset.ratio	-0.050583221	0.420308085
PC6	coronoid.slenderness	0.02322561	0.711500911

Table 1. Table outlining the correlation coefficient of each principal component axis against each jaw trait submitted to the PCA. A *p*-value for the correlation is also recorded.

	Jaw with Most Negative PC Score	Jaw with Most Positive PC Score
PC1	***Hypisodus minimus*, Eocene**	***Megazostrodon rudnerae*, Early Jurassic**
	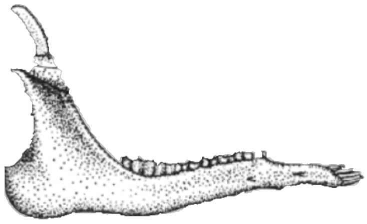	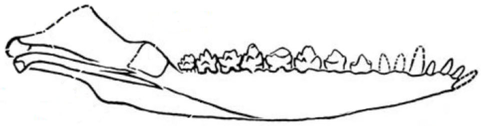
PC2	***Metacheiromys dasypus*, Eocene**	***Hypisodus minimus*, Eocene**
	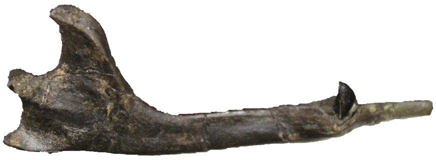	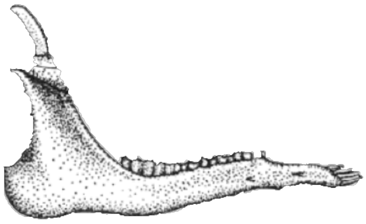
PC3	***Wortmania otariidens*, Paleocene**	***Metacheiromys dasypus*, Eocene**
	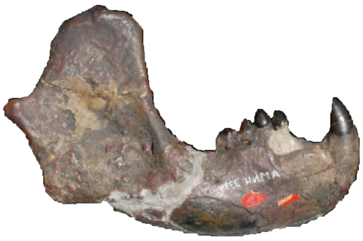	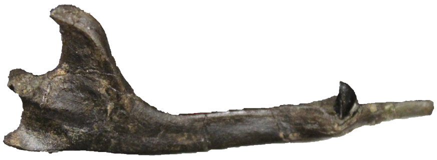
PC4	***Hypisodus minimus*, Eocene**	***Duchesneodus uintensis*, Eocene**
	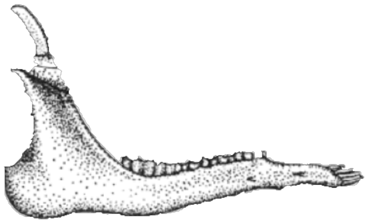	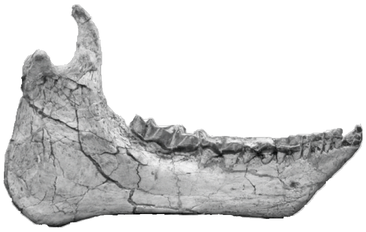
PC5	***Convallisodon convexus*, Paleocene**	***Tillodon fodiens*, Eocene**
	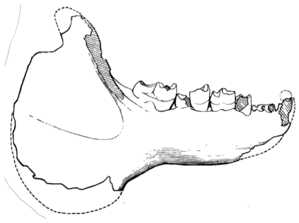	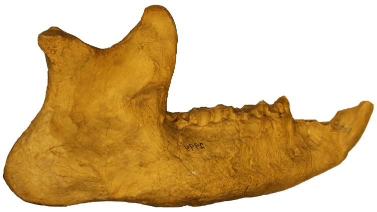
PC6	***Paroodectes feisti*, Eocene**	***Metacheiromys dasypus*, Eocene**
	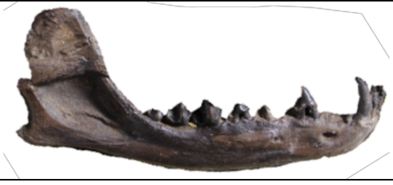	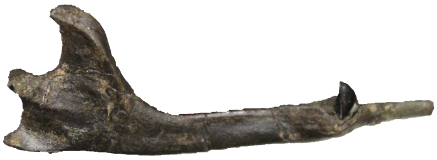

Table 2. Table showing the extreme morphologies of the species that occupy the most extreme points recorded on each principal component axis. *Hypisodus minimus*; sketch of specimen KUVP 127574, edited from Meehan & Martin [1]. *Megazostrodon rudnerae*; reconstruction of specimen BMNH 26407, edited from Kielan-Jaworowska et al [2]. *Metacheiromys dasypus*; photograph of specimen AMNH 11718 taken by GLB. *Wortmania otariidens*; photograph of AMNH 3394, taken from Williamson & Brusatte [3]. *Duchesneodus uintensis*; photograph of CMNH 11809, taken from Mihlbachler [4]. *Convallisodon convexus*; reconstructive sketch of IVPP V5485, taken from Chow & Qi [5]. *Tillodon fodiens*; photograph of CM 2994 (cast) taken by GLB. *Paroodectes feisti*; photograph of jaw of HLMD-Me 7951 (cast) taken by GLB.

References

1. Meehan TJ, Martin LD. 2004 Emended genus description and a new species of *Hypisodus* (Artiodactyla: Ruminantia: Hypertragulidae). *Paleogene Mammals, New Mexico Museum of Natural History and Science Bulletin No. 26*, eds Lucas SG, Zeigler KE, Kondrashov PE (Authority of the State of New Mexico), pp 137-143.

2. Kielan-Jaworowska Z, Cifelli RL, Luo Z-X. 2004 *Mammals from the Age of Dinosaurs: Origins, Evolution, and Structure* New York: Columbia Univ. Press

3. Williamson TE, Brusatte SL. 2013 New Specimens of the Rare Taeniodont Wortmania (Mammalia: Eutheria) from the San Juan Basin of New Mexico and Comments on the Phylogeny and Functional Morphology of "Archaic" Mammals. *PLoS One* **8**, 1-35. (doi:10.1371/journal.pone.0075886)

4. Mihlbachler MC. 2008 Species Taxonomy, Phylogeny, and Biogeography of the Brontotheriidae (Mammalia: Perissodactyla). *Bull. Am. Mus. Nat. Hist* **311**, 1-475.

5. Chow M, Qi T. 1978 Paleocene Mammalian Fossils from Nomogen Formation of Inner Mongolia. *Vertebrata PalAsiatica* **16**, 77-85.

Supplementary Results and Discussion

All five disparity metrics (minimum spanning tree, sum of variance, sum of ranges, mean pairwise distance, mean distance from centroid) show that Cenozoic mammal disparity surpassed Mesozoic mammal disparity by the middle – late Eocene at the latest (Fig. S6a-d). Further, all metrics show an increase in therian mammal disparity across the K/Pg boundary, and a continued increase across the Paleocene/Eocene boundary and throughout the Eocene (Fig. S6a-d). Discrepancies in the time taken for disparity to exceed pre-K/Pg levels reflect the different aspects of spread captured by these metrics. Sum of ranges remains relatively low throughout the Mesozoic and across the K/Pg boundary, but rises quickly across the Paleocene/Eocene boundary and throughout the Eocene. A longer recovery period for the mean distance from centroid suggests a higher proportion of mammals with morphologies close to the centre of function space in the early part of the Cenozoic. Taken together, these results indicate that although the total range of occupied function space increased, the proportion of the sampled population exhibiting these extreme morphologies was relatively low.

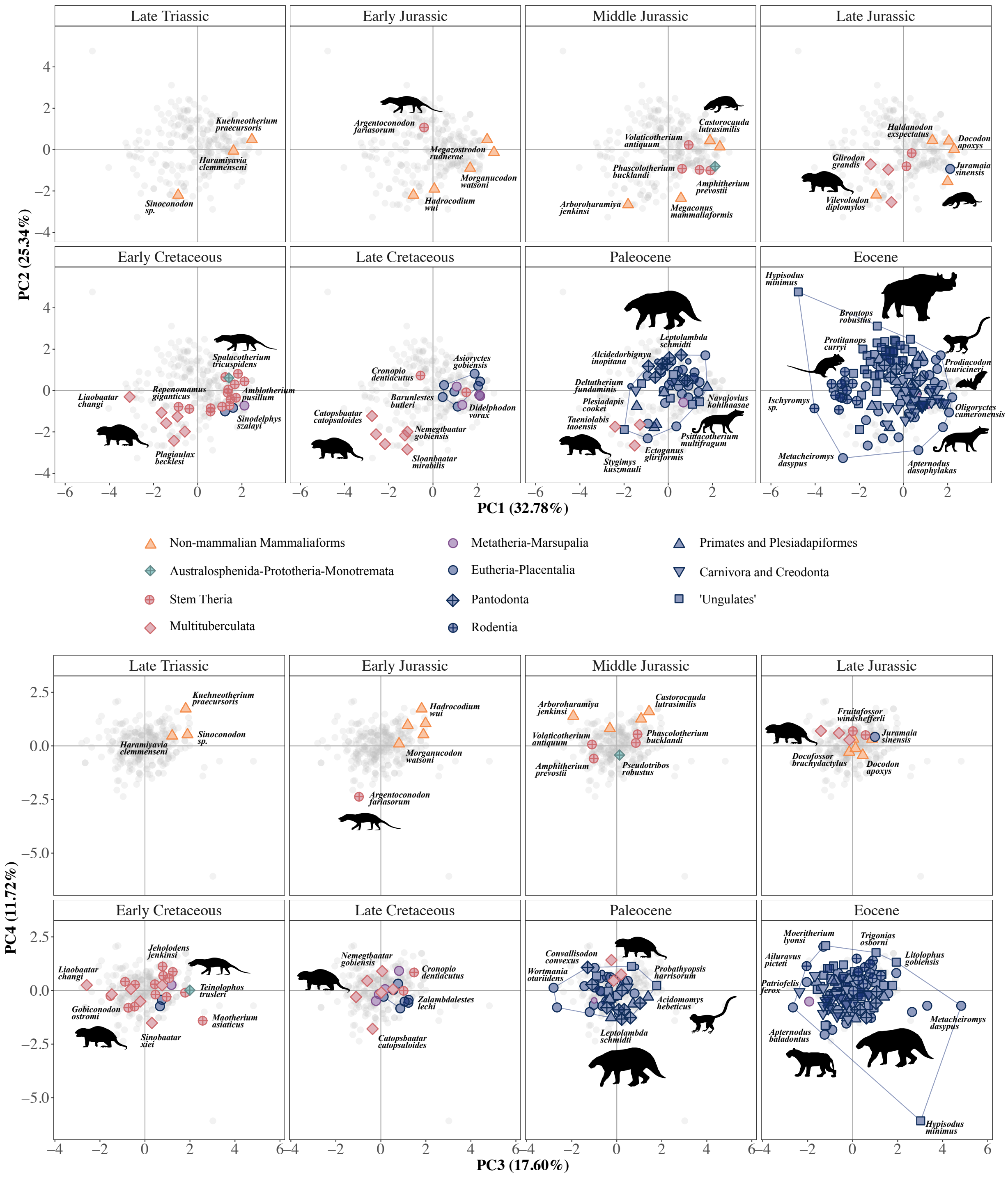

Figure S1. Mammaliaform jaw function space from the Late Triassic – Eocene split between (a) PC1-PC2 and (b) PC3-PC4. Symbol colour and shape represent taxonomic or ecological groupings (see key within figure), and mammals plotting in extreme PC1-4 function space are labelled. Specimens of uncertain age are plotted as full-size symbols in the most likely bin, and at smaller sizes in other possible—but less likely—intervals. The blue polygon depicts the total spread of eutherian and placental mammals in the Late Cretaceous, Paleocene, and Eocene. The grey points represent the spread of all data points across the total study time interval.

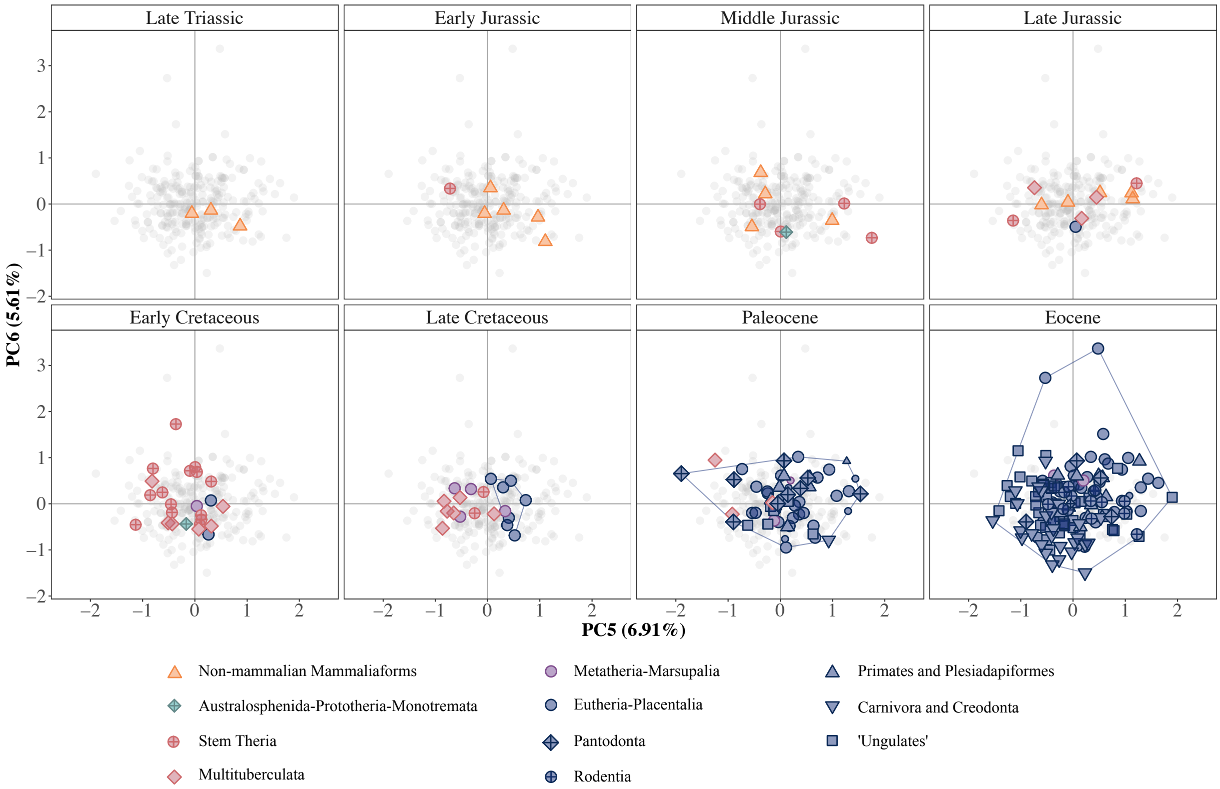

Figure S2. Mammaliaform jaw function space from the Late Triassic – Eocene, for PC5-6. Symbol colour and shape represent taxonomic or ecological groupings (see key within figure). Specimens of uncertain age are plotted as full-size symbols in the most likely bin, and at smaller sizes in other possible—but less likely—intervals. The blue polygon depicts the total spread of eutherian and placental mammals in the Late Cretaceous, Paleocene, and Eocene. The grey points represent the spread of all data points across the total time interval examined.

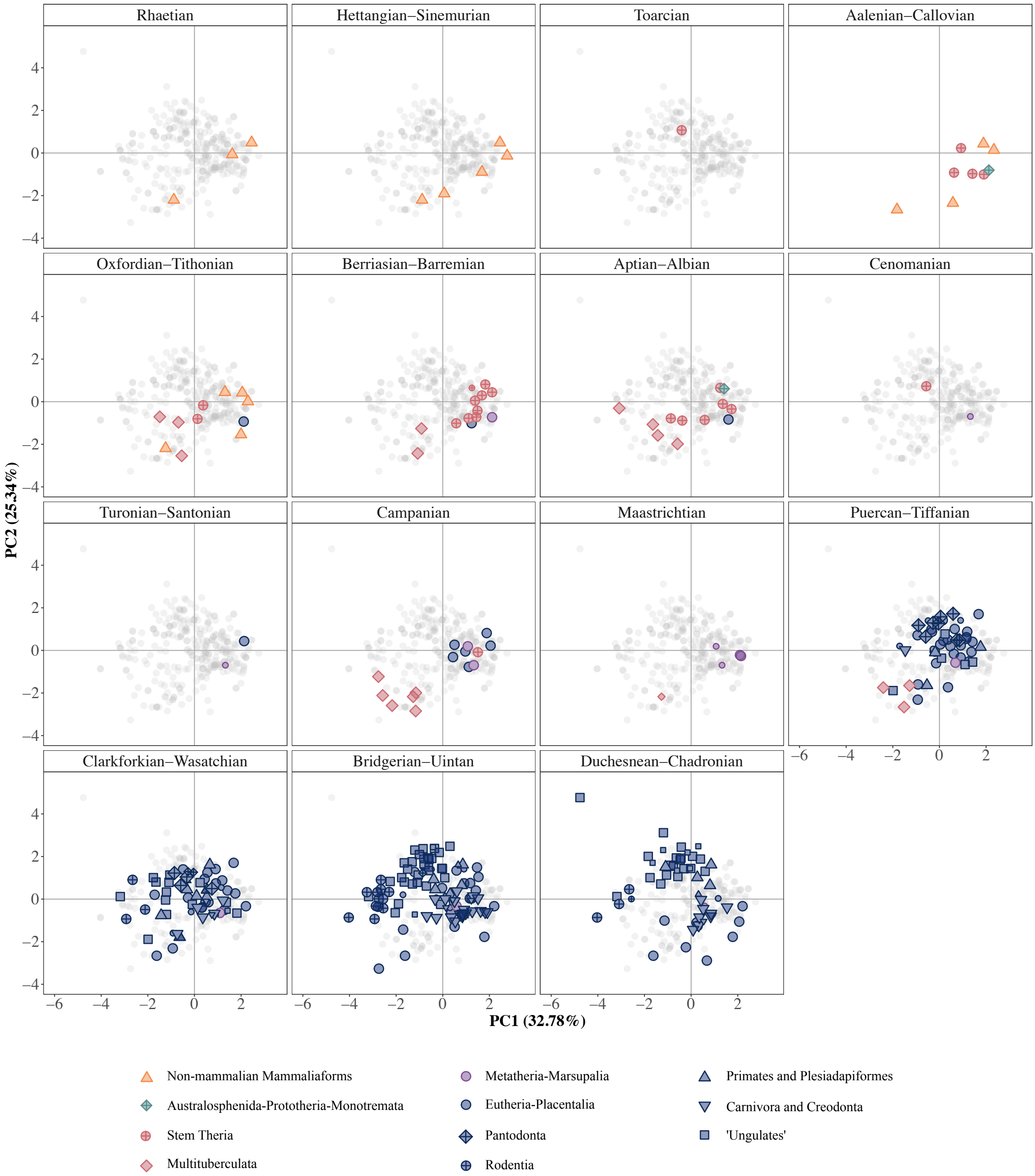

Figure S3. PC1-PC2 Jaw function space across 15 time bins, based on amalgamated stages (Mesozoic) and North American Land Mammal Ages (NALMA; Cenozoic) from the Rhaetian (Late Triassic) – Duchesnean-Chadronian (Eocene). Different colours and symbols represent mammal clades (see key within figure). Specimens of uncertain age are plotted as full-size symbols in the most likely bin, and at smaller sizes in other possible—but less likely—intervals. The grey points represent the spread of all data points across the total study time interval.

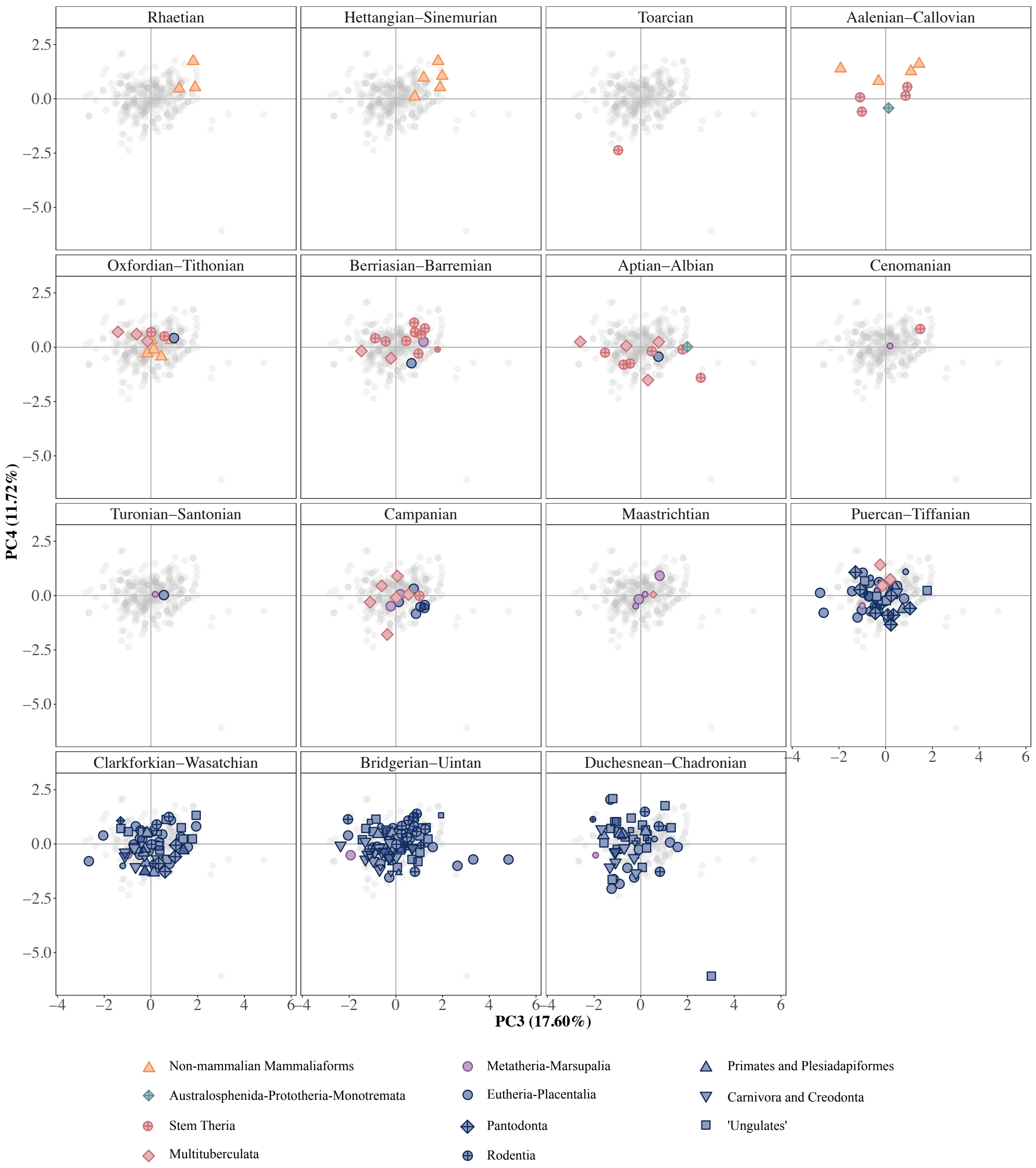

Figure S4. PC3-PC4 Jaw function space across 15 time bins, based on amalgamated stages (Mesozoic) and North American Land Mammal Ages (NALMA; Cenozoic) from the Rhaetian (Late Triassic) – Duchesnean-Chadronian (Eocene). Different colours and symbols represent mammal clades (see key within figure). Specimens of uncertain age are plotted as full-size symbols in the most likely bin, and at smaller sizes in other possible—but less likely—intervals. The grey points represent the spread of all data points across the total study time interval.

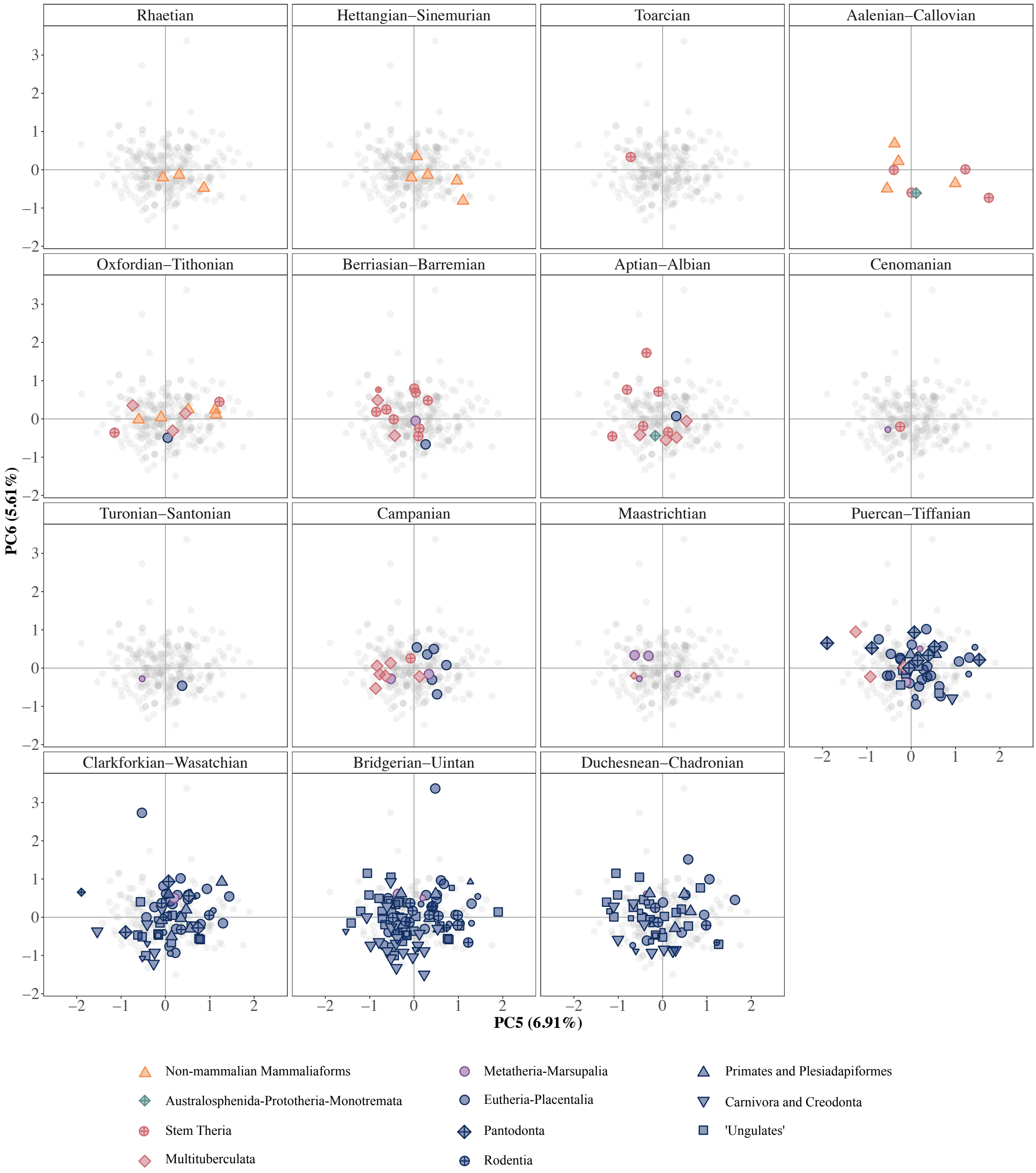

Figure S5. PC5-PC6 Jaw function space across 15 time bins, based on amalgamated stages (Mesozoic) and North American Land Mammal Ages (NALMA; Cenozoic) from the Rhaetian (Late Triassic) – Duchesnean-Chadronian (Eocene). Different colours and symbols represent mammal clades (see key within figure). Specimens of uncertain age are plotted as full-size symbols in the most likely bin, and at smaller sizes in other possible—but less likely—intervals. The grey points represent the spread of all data points across the total study time interval.

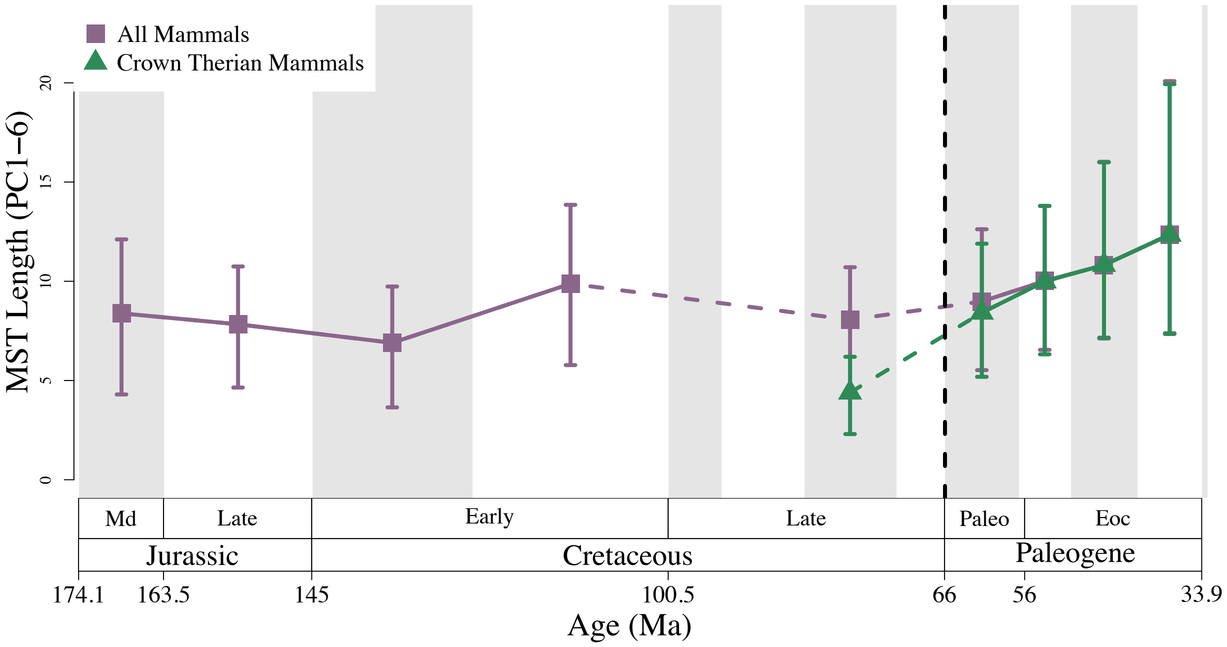

Figure S6. Mammaliaform jaw functional minimum spanning tree (MST) length through time, calculated without the use of grid cells. The purple curve represents all mammals, and the green curve represents therian mammals only. Amalgamated stages (Mesozoic) and NALMAs (Cenozoic) with six or more individuals are used as time bins (see methods). Data is rarefied (n = 6) and sampled with replacement to enable fairer comparison between bins with differing sample sizes. The 95% confidence intervals represent uncertainty estimated by resampling procedure described in text. A solid line indicates changes between consecutive time bins. Dashed lines indicate changes across two or more time bin boundaries.

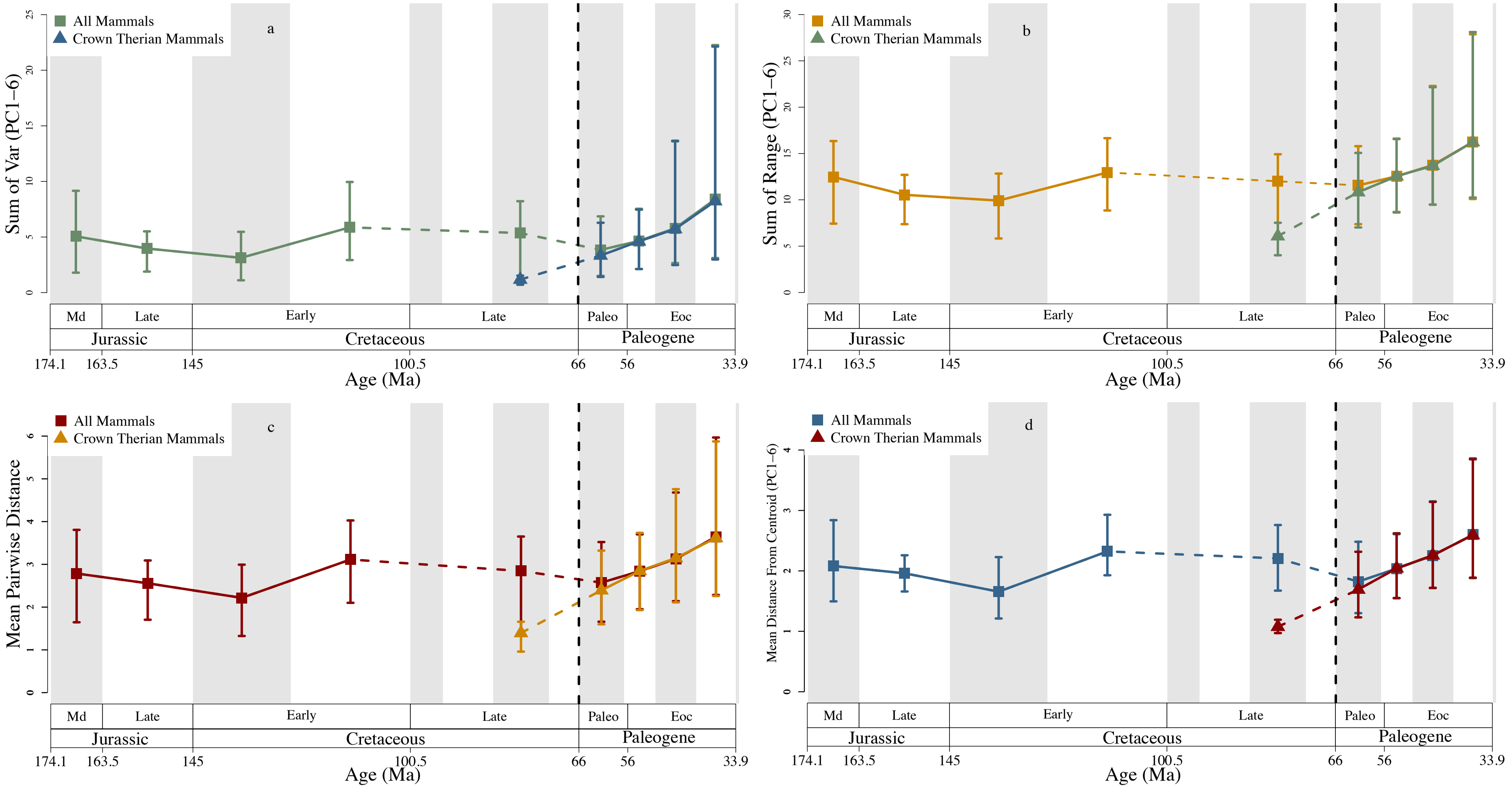
All Mammals
Crown Therian Mammals
a
Sum of Var (PC1−6)
b
Sum of Range (PC1−6)
c
Mean Pairwise Distance
d
Mean Distance From Centroid (PC1−6)
Md
Late
Early
Late
Paleo
Eoc
Jurassic
Cretaceous
Paleogene
174.1
163.5
145
100.5
66
56
33.9
Age (Ma)

Figure S7. Jaw functional disparity through time, calculated using four different methods. The four methods shown here are a) sum of variance, with the green curve showing sum of variance among all mammals, and the blue showing therian mammals only, b) sum of ranges, with the orange curve showing sum of ranges among all mammals, and the green showing therian mammals only c) mean pairwise distance, with the red curve showing all mammals, and the orange showing therian mammals only and d) mean distance from centroid, with the blue curve showing all mammals, and the red showing therian mammals only. For all methods (a-d), amalgamated stages (Mesozoic) and NALMAs (Cenozoic) with six or more individuals are used as time bins (see methods). Data is rarefied (n = 6) and sampled with replacement to enable fairer comparison between bins with differing sample sizes. The 95% confidence intervals represent uncertainty produced by sampling six individuals at random with replacement across each time bin, for 5000 iterations. The points represent the mean values of these 5000 runs. A solid line indicates changes between consecutive time bins. Dashed lines indicate changes across two or more time bin boundaries. Although these curves represent rarefaction to six, total mammal disparity through time has been rarefied to fifteen (across fewer bins) across all methods and can be seen in Fig. S7b-e. Analysis using this higher rarefaction number recovers the same patterns across the K/Pg boundary and into the Cenozoic for all four methods.

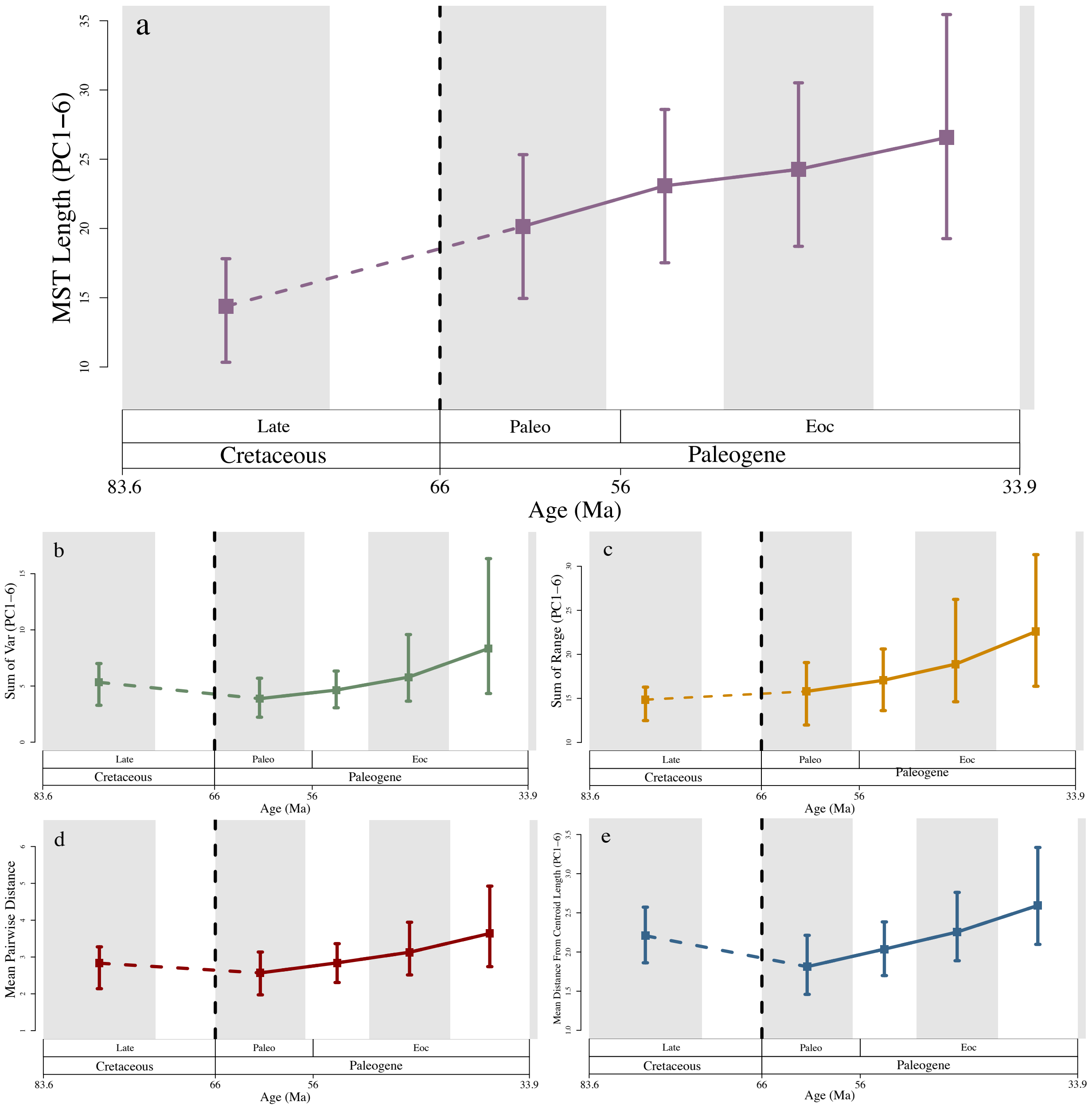

Figure S8. Jaw functional disparity among all mammals across the K/Pg boundary, calculated using five different methods. The four methods shown here are a) minimum spanning tree b) sum of variance, c) sum of ranges, d) mean pairwise distance, and e) mean distance from centroid. For all methods (a-e), the Campanian stage (Mesozoic) and NALMAs (Cenozoic) are used as time bins (see Material and methods). For each disparity method, data is rarefied (*n* = 15) and sampled with replacement to enable fairer comparison between bins with differing sample sizes. The 95% confidence intervals represent uncertainty produced by sampling fifteen individuals at random with replacement across each time bin, for 5000 iterations. The points represent the mean values of these 5000 runs. A solid line indicates changes between consecutive time bins. Dashed lines indicate changes across two or more time bin boundaries.
